# Arc Fault Detection for Photovoltaic Systems Using Independent Component Analysis Technique and Dynamic Time-Warping Algorithm

**DOI:** 10.3390/s25196094

**Published:** 2025-10-03

**Authors:** Jiazi Xu, Shuo Ding, Guoli Li, Qunjing Wang

**Affiliations:** 1 School of Electronic and Information Engineering, Anhui University, Hefei 230601, China; xjz303@ahu.edu.cn; 2 School of Electrical Engineering and Automation, Anhui University, Hefei 230601, China; z24301259@stu.ahu.edu.cn (S.D.); liguoli@ahu.edu.cn (G.L.)

**Keywords:** photovoltaic system, arc faults, global feature, independent component analysis, dynamic time warping

## Abstract

Arc fault detection in photovoltaic systems is crucial, since it may cause incidents like fires and explosions. So far, most existing methods rely on an arc’s local features and do not characterize arc faults globally, which may lead to detection failure in noisy environments. In this paper, a fundamentally different method is proposed that relies on an arc’s global features instead of local ones. The core idea of the method is that the physical mechanisms of the arc fault signals and the normal signals are so different that they are thought to be generated by two independent sources. Based on this insight, independent component analysis (ICA) is introduced to decompose the photovoltaic system’s DC currents. By using ICA, the DC current signals with an arc fault can be decomposed into two independent signals, while the normal signals without an arc fault cannot be decomposed into two such independent signals. This indicates that arc faults can be detected by using the concept of “independence”. Then, the dynamic time warping algorithm was used to determine the independence level of the ICA outputs so as to realize end-to-end arc fault detection. Experimental results showed that our method has better performance than traditional methods in terms of detection accuracy and robustness against environmental disturbances.

## 1. Introduction

Arc fault detection in photovoltaic (PV) systems is crucial, since it may cause incidents like fires and explosions [1,2,3,4]. In general, arc faults can be classified into three categories as follows: (1) series arcs, which appear at the break point of a conductor or the bad connection point at the DC side [5]; (2) parallel arcs, which may occur between conductors with different potentials; and (3) ground arcs, which may occur when a current path is formed through the ground [6,7]. Among the above categories, series arc fault detection is the most challenging task because an arc signal is too small to be easily detected, which has led to extensive research on it [8,9,10,11].

So far, most of the series arc fault detection methods can be divided into two categories, namely feature-based and data-based methods. Feature-based methods mainly detect the arc features from the time or frequency domain. For instance, F. Schimpf detected arc faults by investigating the variance of arc current signals [12], Qing xiong et al. used pulse amplitude and spectral characteristics to detect arc faults [13], Wang used discrete wavelet transform (DWT) to detect arc faults [14], and Sidhu T.S. et al. used arc infrared and electromagnetic radiation to detect arc faults. These methods have high interpretability and can be easily realized in practical situations. However, these methods can only extract an arc’s local features, which means such features can only describe part of the arc characteristics. These local features have the limitation of low robustness against disturbances and may lead to failure in arc fault detection. For example, some external disturbances, such as power grid disturbance, may have a similar frequency characteristic as arc signals, resulting in possible failure detection when spectral-based methods are used [15].

Another category is data-based detection methods, which typically use machine learning techniques to detect arc faults. For instance, J. A. Momoh R, Dipti D. Patil, and Shibo Lu used classical neural networks [16], convolution neural networks (CNNs) [17], and domain-adaptive and deep convolutional generative adversarial networks (DA-DCGANs) [18] to detect arc faults, respectively. These methods extract an arc’s features from real-world data so as to implement end-to-end detection. However, these methods’ accuracy depends on the quality of data and has the limitations of low interperability and low generalization.

In this paper, a fundamentally different method is proposed, which combines the advantages of both feature-based and data-based methods. The core idea of our method is that the physical mechanism of the arc signals and the normal ones is considered to be so different, that they can be produced by two independent sources. Based on this insight, the PV system’s DC-side current signals were decomposed by using independent component analysis. The results show that the PV system’s DC-side current signals with an arc fault can be decomposed into two independent signals, while the normal signals without an arc fault cannot be decomposed into two such independent signals (ICA). This indicates that the arc fault can be detected by using concept of “independence”. After that, the dynamic time warping (DTW) algorithm was used to determine the independence level of the output of independent component analysis so as to realize robust and end-to-end arc fault detection.

## 2. Background: Arc Characteristics

In this section, the arc characteristics are analyzed from the global feature perspective, and two global characteristics of the arc are illustrated, namely *independence* and *non-Gaussian*. Concretely, *independence* means that the arc fault and the normal signals are independent of each other, and *non-Gaussian* means that the arc fault signals does not obey a Gaussian distribution.

### 2.1. Independence of the Arc Fault Signal

**Figure 1:**
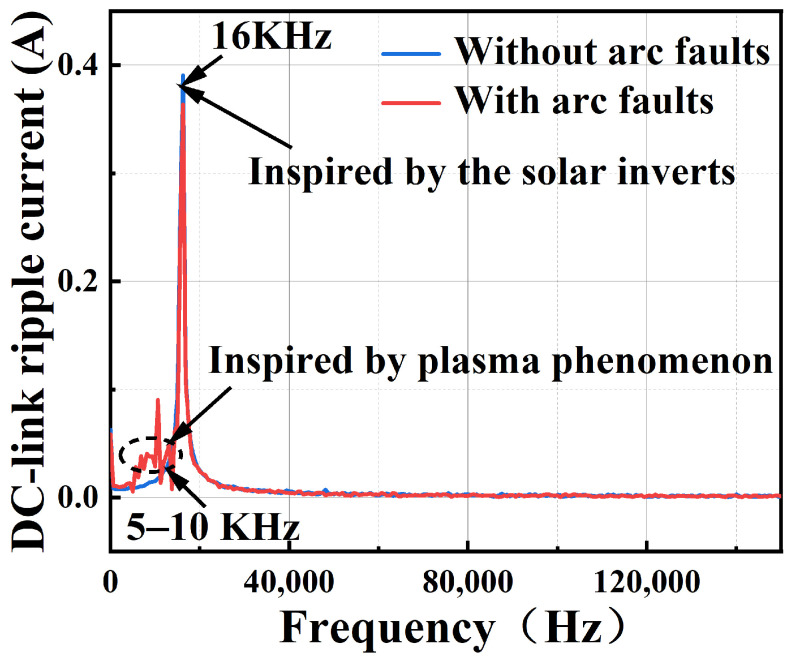
The spectrum of the arc fault signal and the normal signal.

One important global feature of an arc fault is that it is independent of normal signals. In the PV system, the normal DC current signals are inspired by the solar panels, while the arc fault signals are inspired by the plasma phenomenon. This indicates that the physical mechanism of the arc fault and the normal signals are totally different and can be viewed as inspired by independent sources. This can be clarified in Figure 1, whose data is obtained from real-world experiments, where the frequency components of the PV system’s DC-side currents are mainly located in 16 KHz, which is the PWM switching frequency of solar inverts, while the frequency components of arc fault signals are mainly located in 5–10 KHz, as shown by the dashed circle in the Figure 1, which is in line with the principles of randomness of plasma motion.

### 2.2. Non-Gaussianity of the Arc Fault Signal

Another important global feature of the arc fault is that it is non-Gaussian. This can be testified by using kurtosis theory [19]. Mathematically, kurtosis theory can reflect the signal’s distribution characteristics. A distribution with a higher kurtosis value is more likely to be non-Gaussian. Mathematically, given a sequence of random vector $y=[y_{1},y_{2},\ldots ,y_{i}]$, where $y_{i}\in R^{t}$, its kurtosis value can be calculated as

(1) $$\begin{array}{c} kurt\left(y\right)=E\left\{y^{4}\right\}-3{\left(E\left\{y^{2}\right\}\right)}^{2}. \end{array}$$

where E{·} means the expectation of variables.

**Figure 2:**
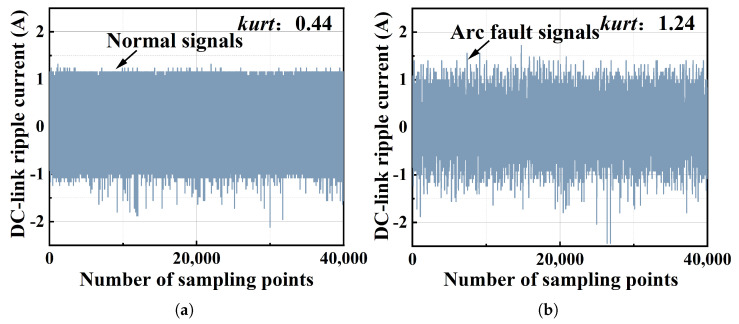
The normal PV system signal and the arc fault signal, in which the sampling rate is 1 MHz. (**a**) The normal PV system signal; the kurtosis value is 0.44. (**b**) The arc fault signal; the kurtosis value is 1.24.

**Figure 3:**
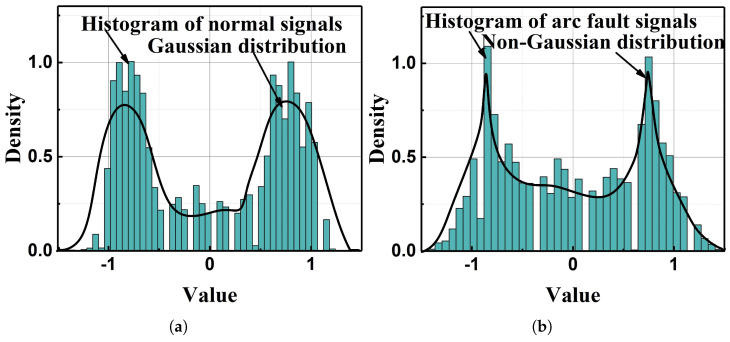
The real distribution of the normal signal and the arc fault signal: (**a**) the normal signal’s real distribution; (**b**) the arc fault signal’s real distribution.

Figure 2 shows the kurtosis value of a normal signal and arc fault signal whose data is obtained from real-world experiments. It can be seen that the kurtosis value of the normal signal is 0.44, while the kurtosis value of the arc signal is 1.24. This indicates that the signal with the arc fault is more likely non-Gaussian. More evidence of the arc fault’s non-Gaussian characteristics is shown in Figure 3 by using histograms, where the normal signal’s distribution is apparently Gaussian, while the arc fault signal’s distribution is non-Gaussian.

## 3. Method

In this study, the concept of independence is intended to be used to detect arc faults. To achieve this, independent component analysis (ICA) is firstly employed to decompose the PV system’s DC current signals, and then the dynamic time warping (DTW) algorithm is used to determine the independent level of the ICA’s output.

### 3.1. Using ICA Technique to Decompose Signals

The ICA technique [20] is introduced to decompose the observed signals. Given *n* sets of independent signals $s={\left[s_{1},s_{2},\ldots ,s_{n}\right]}^{T}$, where $s_{i}\in R^{m}$, we have

(2) $$\begin{array}{c} x=As. \end{array}$$

where $x={\left[x_{1},x_{2},\ldots ,x_{n}\right]}^{T},x_{i}\in R^{m}$ is the *n* observed signals that are mixed from ${\left[s_{1},s_{2},\ldots ,s_{n}\right]}^{T}$, $A\in R^{n\times n}$ is an invertible matrix, called the mixing matrix. The goal of ICA is to recover $\hat{s}$ from x, where $\hat{s}={\left[{\hat{s}}_{1},{\hat{s}}_{2},\ldots ,{\hat{s}}_{n}\right]}^{T}$ is an estimate of s. To achieve this, a new matrix W is constructed such that

(3) $$\begin{array}{c} \hat{s}=\mathrm{Wx}. \end{array}$$

**Figure 4:**
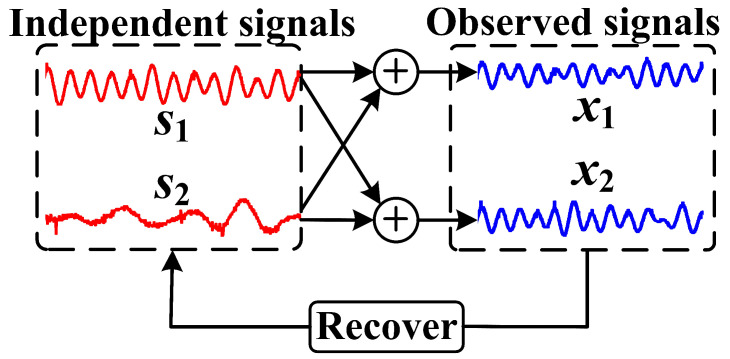
The example of ICA that can recover the independent signals s from the observed signals x, where $s=\{s_{1},s_{2}\}$ comprises two independent sources and $x=\{x_{1},x_{2}\}$ are two observed signals that are mixed from $s_{1},s_{2}$.

Based on Equation (3), the goal of ICA becomes finding an unmixing matrix W that fits the approximation of $A^{-1}$ such that $\hat{s}\approx s$. A brief description is demonstrated in Figure 4.

### 3.2. Brief Description of ICA Technique

This subsection gives a brief description of the ICA technique. Generally, ICA can be achieved through the technique of singular value decomposition (SVD). SVD provides a method for dividing A into several simpler pieces, which is

(4) $$\begin{array}{c} A=U\Sigma V^{T}. \end{array}$$

where U and V are orthogonal matrices and Σ is a diagonal matrix with real, non-negative values. Then

(5) $$\begin{array}{c} W=A^{-1}={\left(V^{T}\right)}^{-1}\Sigma^{-1}U^{-1}=V\Sigma^{-1}U^{T}. \end{array}$$

Thus, W, U, Σ, and V need to be calculated, and this can be divided into two parts as follows: (**1**) signal whitening and (**2**) independent signal separating.

**Signal whitening** uses a set of linear transformations that can flip and scale the signals to transform the signals into normalized ones. We can perform whitening on x, that is

(6) $$\begin{array}{c} x_{w}=\left(D^{-\frac{1}{2}}E^{T}\right)x \end{array}$$

where $x_{w}$ is a whitening of x; E is an orthogonal matrix, whose columns are the eigenvectors of the covariance of x; and D is a diagonal matrix of the eigenvalues, and each diagonal entry in D is an eigenvalue of the covariance of x. The covariance of $x_{w}$ is written as $\langle x_{w}x_{w}^{T}\rangle =I$, where $\langle \cdot \rangle$ means the covariance of variables and I is the unit matrix.

Then, we can use the signal whitening to calculate U and Σ. Assuming s is whitened, that is, $\langle ss^{T}\rangle =I$, we can obtain

(7) $$\begin{array}{cc} \langle xx^{T}\rangle & =\langle \left(\mathrm{As}\right){\left(\mathrm{As}\right)}^{T}\rangle \\ & =\langle \left(U\Sigma V^{T}s\right){\left(U\Sigma V^{T}s\right)}^{T}\rangle \\ & =U\Sigma^{2}U^{T}. \end{array}$$

where $\langle xx^{T}\rangle$ is the covariance of x, and it also can be expressed as

(8) $$\begin{array}{c} \langle {\mathrm{xx}}^{T}\rangle ={\mathrm{EDE}}^{T}. \end{array}$$

Combining Equations (7) and (8), there are

(9) $$\begin{array}{c} \left\{\begin{array}{c} U=E\\ \Sigma =D^{\frac{1}{2}} \end{array}\right. \end{array}$$

According to calculations of U and Σ,

(10) $$\begin{array}{c} \hat{s}=Wx=V\Sigma^{-1}U^{T}x={\mathrm{VD}}^{-\frac{1}{2}}E^{T}x=Vx_{w}. \end{array}$$

where V is the only unknown matrix that needs to be calculated.

**Independent signal separating** aims to find the optimal solution of matrix V that can make $\hat{s}$ independent. To solve this issue, mutual information [21,22] is used to evaluate the independence of $\hat{s}$. For $\hat{s}={\left[{\hat{s}}_{1},{\hat{s}}_{2},\ldots ,{\hat{s}}_{n}\right]}^{T}\in R^{n\times t}$, if each column of $\hat{s}$ is independent, their mutual information should be zero, that is [21],

(11) $$\begin{array}{c} I\left(\hat{s}\right)=\int p\left(\hat{s}\right)\log_{2}\frac{p(\hat{s})}{\prod_{i}p\left({\hat{s}}_{i}\right)}d\hat{s}=0. \end{array}$$

where $I(\hat{s})$ is the mutual information of $\hat{s}$; $p(\hat{s})$ is the joint distribution of $\hat{s}$. The mutual information $I(\hat{s})$ represents the difference between the entropy of the marginal distributions and the entropy of the joint distribution, which is

(12) $$\begin{array}{cc} I(\hat{s}) & =\sum_{i}H\left[{\hat{s}}_{i}\right]-H\left[\hat{s}\right]\\ \; & =\sum_{i}H\left[{\left(Vx_{w}\right)}_{i}\right]-H\left[Vx_{w}\right]\\ \; & =\sum_{i}H\left[{\left(Vx_{w}\right)}_{i}\right]-\left(H\left[x_{w}\right]+\log_{2}\left|V\right|\right). \end{array}$$

where $H\left[\cdot \right]$ is entropy, according to Equation (4), that is, $\log_{2}\left|V\right|$ = 0, making $H[x_{w}]$ a constant and independent of V. Combining Equations (11) and (12), V can be calculated as

(13) $$\begin{array}{c} V=\underset{V}{\arg\min}\sum_{i}H\left[{\left(Vx_{w}\right)}_{i}\right]. \end{array}$$

**Figure 5:**
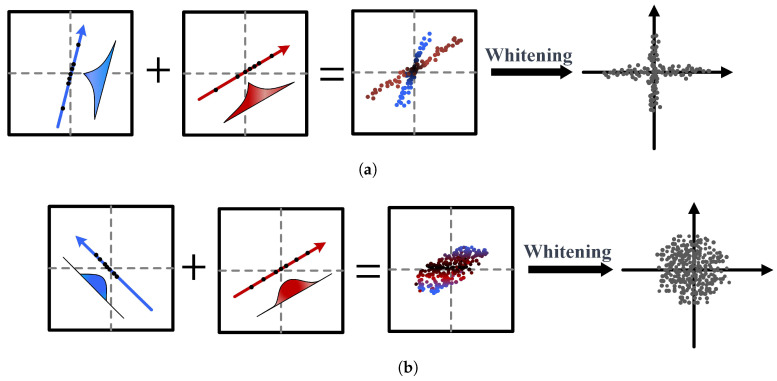
Schematic diagram of applying ICA to decompose Gaussian and non-Gaussian signals: (**a**) two non-Gaussian signals can be separated. (**b**) Two Gaussian signals cannot be separated.

It should be noted that the ICA technique can only decompose non-Gaussian signals, as shown in Figure 5. Specifically, Figure 5a shows that if two non-Gaussian signals are superimposed, the signal whitening indicates that they can be separated by signal whitening. Figure 5b shows that if two Gaussian signals are superimposed, they are overlapped and cannot be separated by signal whitening.

### 3.3. The Reason Why ICA Can Be Used for Arc Fault Detection

In this subsection, the key reasons why ICA can be used for arc fault detection are revealed. It is shown that if ICA is used, the DC current signal with the arc fault can be decomposed into two independent signals, while the normal signal without an arc fault cannot be decomposed into such two independent signals; the proof is as follows:

**ICA for arc fault signals:** Assume that the observed arc fault signals are $x_{a}={\left[x_{a1},x_{a2}\right]}^{T}$, where $x_{a1}$ and $x_{a2}$ mean the DC-side currents are collected at different moments. According to the analysis in Section 1, $x_{a1}$ and $x_{a2}$ are mixed from independent signals $s_{a1}$ and $s_{a2}$, written as $x_{a1}=c_{1}s_{a1}+c_{2}s_{a2}$ and $x_{a2}=c_{3}s_{a1}+c_{4}s_{a2}$, where $c_{1}$, $c_{2}$, $c_{3}$, and $c_{4}$ are constants. Because $s_{a1}$ and $s_{a2}$ are independent, we have $p\left(s_{a1},s_{a2}\right)=p\left(s_{a1}\right)p\left(s_{a2}\right)$, where $p(s_{a1},s_{a2})$ is the joint probability density of $s_{a1}$ and $s_{a2}$, $p(s_{a1})$ is the probability density of $s_{a1}$, and $p(s_{a2})$ is the probability density of $s_{a2}$. Thus, the mutual information of $s_{a1},s_{a2}$ is

(14) $$\begin{array}{c} \begin{array}{cc} I\left(s_{a1},s_{a2}\right) & =\;\int \int \;p\left(s_{a1},s_{a2}\right)\log_{2}\frac{p\left(s_{a1},s_{a2}\right)}{p\left(s_{a1}\right)p\left(s_{a2}\right)}ds_{a1}ds_{a2}\\ \; & =\;\int \int \;p\left(s_{a1},s_{a2}\right)\log_{2}1ds_{a1}ds_{a2}=0. \end{array} \end{array}$$

Based on Equations (11) and (14), $\left\{s_{a1},s_{a2}\right\}$ are the solutions of ICA.

**ICA for normal signals:** Assuming the observed normal signals $x_{b}={\left[x_{b1},x_{b2}\right]}^{T}$, $x_{b1}$ and $x_{b2}$ are the DC-side currents and are collected at two different moments. According to the analysis in Section 3, $x_{b1}$ and $x_{b2}$ are the linear transformation of $s_{b}$, written as $x_{b1}=c_{5}s_{b}$$x_{b2}=c_{6}s_{b}$, where $c_{5}$ and $c_{6}$ are constants. Now, inputting $x_{b}$ to ICA, there are

(15) $$\left[\begin{array}{c} {\hat{s}}_{b1}\\ {\hat{s}}_{b2} \end{array}\right]=W\left[\begin{array}{c} c_{5}s_{b}\\ c_{6}s_{b} \end{array}\right]$$

where ${\hat{s}}_{b}={\left[{\hat{s}}_{b1},{\hat{s}}_{b2}\right]}^{T}$ is the ICA output.

Equation (15) indicates that ${\hat{s}}_{b1}$, ${\hat{s}}_{b2}$ are the linear transformations of $s_{b}$, so we have $p({\hat{s}}_{b1}$, ${\hat{s}}_{b2})=p\left(s_{b}\right)$, where $p(s_{b1},s_{b2})$ is the joint probability density of $s_{b1}$ and $s_{b2}$ and $p(s_{b})$ is the probability density of $s_{b}$. Thus, the mutual information of ${\hat{s}}_{b1}$, ${\hat{s}}_{b2}$ is

(16) $$\begin{array}{c} \begin{array}{cc} I\left({\hat{s}}_{b1},{\hat{s}}_{b2}\right) & =\;\int \int \;p\left({\hat{s}}_{b1},{\hat{s}}_{b2}\right)\log_{2}\frac{p\left({\hat{s}}_{b1},{\hat{s}}_{b2}\right)}{p\left({\hat{s}}_{b1}\right)p\left({\hat{s}}_{b2}\right)}d{\hat{s}}_{b1}d{\hat{s}}_{b2}\\ \; & =\int p\left(s_{b}\right)\log_{2}\frac{p(s_{b})}{p\left({\hat{s}}_{b1}\right)p\left({\hat{s}}_{b2}\right)}ds\\ \; & =-\log_{2}p\left({\hat{s}}_{b1}\right)p\left({\hat{s}}_{b2}\right)\cdot \int p\left(s_{b}\right)\log_{2}p\left(s_{b}\right)ds_{b}. \end{array} \end{array}$$

Since $p\left({\hat{s}}_{b1}\right),p\left({\hat{s}}_{b2}\right)$ are constant, the entropy of $s_{b}$ is

(17) $$\begin{array}{c} H\left(s_{b}\right)=-\int p\left(s_{b}\right)\log_{2}p\left(s_{b}\right)ds_{b}. \end{array}$$

Combining Equations (16) and (17), we have

(18) $$\begin{array}{cc} I\left({\hat{s}}_{b1},{\hat{s}}_{b2}\right) & =\log_{2}p\left({\hat{s}}_{b1}\right)p\left({\hat{s}}_{b2}\right)\cdot H\left(s_{b}\right)\\ \; & \propto H\left(s_{b}\right)\neq 0. \end{array}$$

Equation (18) indicates that ${\hat{s}}_{b1}$ and ${\hat{s}}_{b2}$ are not independent. Therefore, ICA cannot decompose the normal signal into two independent signals.

### 3.4. Using DTW to Determine the Independence Level of ICA Output

After the calculation of ICA, the DTW algorithm [23,24,25] is used to determine the independent level of ICA output to achieve end-to-end arc fault detection. The reason DTW was chosen is its ability to handle temporal distortions and phase shifts in current signals. The detailed steps of DTW are

**Step1: Signal normalization:** Given the ICA output $\hat{s}={\left[{\hat{s}}_{1},{\hat{s}}_{2}\right]}^{T}$, $\hat{s}$ is normalized as

(19) $$\begin{array}{c} {\hat{s}}_{i}^{\prime }=\frac{{\hat{j}}_{i}-\mu \left({\hat{s}}_{i}\right)}{\sigma \left({\hat{s}}_{i}\right)}. \end{array}$$

where ${\hat{s}}_{i}$ is the $i_{th}$ component of $\hat{s}$, $u({\hat{s}}_{i})$ and $\sigma ({\hat{s}}_{i})$ are the mean and standard deviation of ${\hat{s}}_{i}$, and ${\hat{s}}_{i}^{\prime }$ is the normalized form of ${\hat{s}}_{i}$.

**Step2: Distance calculation:** According to Equation (20), we calculate the DTW absolute distance between ${\hat{s}}_{1}^{\prime }=\left[{\hat{s}}_{11}^{\prime },{\hat{s}}_{12}^{\prime },\ldots ,{\hat{s}}_{1k}^{\prime },\ldots ,{\hat{s}}_{1m}^{\prime }\right]$ and ${\hat{s}}_{2}^{\prime }=\left[{\hat{s}}_{21}^{\prime },{\hat{s}}_{22}^{\prime },\ldots ,{\hat{s}}_{2l}^{\prime },\ldots ,{\hat{s}}_{2m}^{\prime }\right]$ as

(20) $$\begin{array}{c} \left\{\begin{array}{c} D(1,1)=d(1,1)\\ D(1,l)=d(1,l)+D(1,l-1)\\ D(k,1)=d(k,1)+D(i-1,1)\\ D(k,l)=d(k-1,l-1)+\min[D(i,l-1),\\ D(i-1,l),D(i-1,l-1) \end{array}\right. \end{array}$$

where d(k,l) is the Euclidean distance between ${\hat{s}}_{1k}^{\prime }$ and ${\hat{s}}_{2l}^{\prime }$:

(21) $$\begin{array}{c} d\left(k,l\right)=\sqrt{{\left({\hat{s}}_{1k}^{\prime }-{\hat{s}}_{2l}^{\prime }\right)}^{2}}. \end{array}$$

D(k,l) is the minimum absolute distance we find from d(1,1) to d(k,l).

## 4. Experiment

### 4.1. Experimental Platform

**Figure 6:**
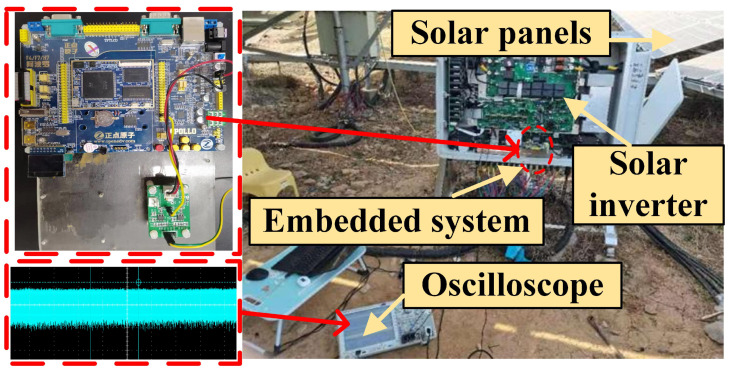
The actual PV system experimental platform.

A real-world PV platform is used for performance evaluation. The experimental rig is shown in Figure 6, which consists of a solar inverter for signal generation, an embedded system for signal collection, and an oscilloscope for signal presentation. The solar inverter’s maximum DC voltage is 1500 V, and the maximum DC current is 20 A. The DC-side current is measured by a Hall-effect current sensor manufactured by Sinomags Technology in Bengbu, Anhui, China. The acquired current signals are then transmitted to a microcontroller, where they are converted into digital values by its built-in analog-to-digital converter (ADC). The ADC operates at a conversion rate of 1 Mbps, meaning that one million analog-to-digital conversions are performed per second. In this study, the solar inverter’s DC-side current signals are used and collected to detect DC-side arc faults. The detailed steps are shown below.

**Figure 7:**
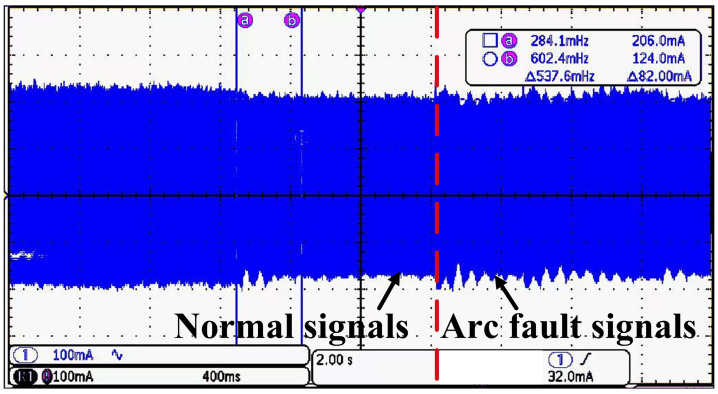
Oscilloscope presentation image.

**Step 1—Signal generation and collection:** The current signals under both arc fault and normal scenarios are collected, respectively, which are shown using an oscilloscope in Figure 7. An embedded system is used for signal collection and calculation. The core MCU of the embedded system is STM32H743ZIT6, and the sampling rate is 1 Mbps.

**Step 2—ICA and DTW calculation:** After signal collection, the data is sent to STM32H743ZIT6 for calculation. The algorithm of ICA and DTW are realized by using X-CUBE-AI and STM32CubeMX, which are the libraries of STM32-MCU.

### 4.2. Experiment Detection Results

**Figure 8:**
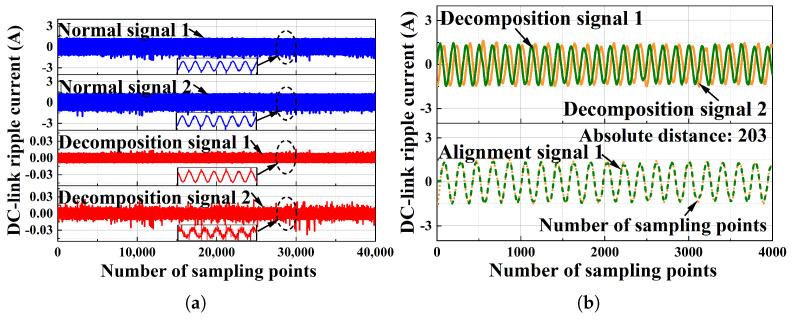
The detection results of the normal signal when the PV system DC is 6 A: (**a**) the results of the ICA output when the PV system DC is 6 A; (**b**) the results of DTW when the PV system DC is 6 A.

**Figure 9:**
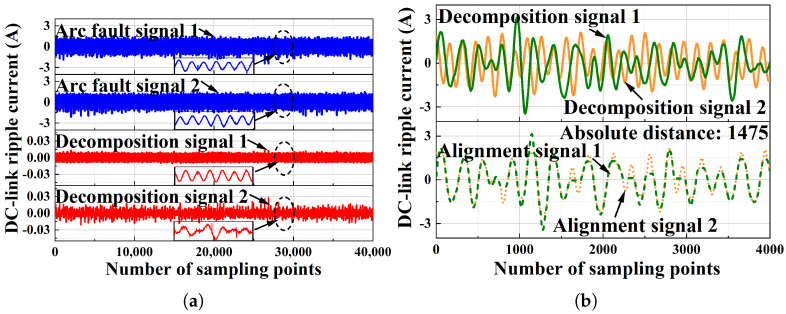
The detection results of the arc fault signal when the PV system DC is 6 A: (**a**) the results of DTW when the PV system DC is 6 A; (**b**) the results of DTW when the PV system DC is 6 A.

The effectiveness of the proposed method is evaluated at a DC-side current of 6 A. The normal signal results are shown in Figure 8, where Figure 8a shows the results of the ICA output, and the DTW distance is shown in Figure 8b. The arc fault signals’ experimental results are shown in Figure 9, where Figure 9a shows the results of the ICA output, and the DTW distance is shown in Figure 9b. The results for both the normal signals and the arc fault signals were obtained from real-world experiments, and the number of sampling points is 40,000 for ICA and 4000 for DTW. It can be seen in Figure 8 and Figure 9 that the normal signals’ absolute distance is 203, and the arc fault signals’ absolute distance is 1475. This indicates that the arc fault can be easily detected by setting a proper threshold for the DTW distance value.

**Figure 10:**
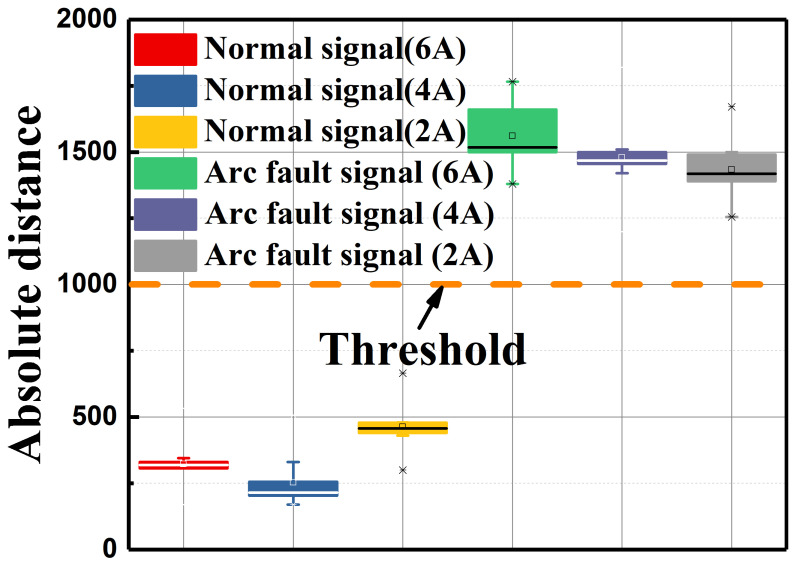
Experimental results at different currents.

In order to determine the proper DTW distance threshold under different current levels, 50 sets of experiments were repeated at 6A, 4 A, and 2 A DC-side currents, respectively. The box plot is shown in Figure 10, where the normal signals’ DTW distance is between 100 and 400 and the arc fault signals’ DTW distance is between 1200 and 1800. Thus, in this study, we set the DTW distance to 1000 as an empirical threshold.

### 4.3. Experiment in Noisy Environment

**Figure 11:**
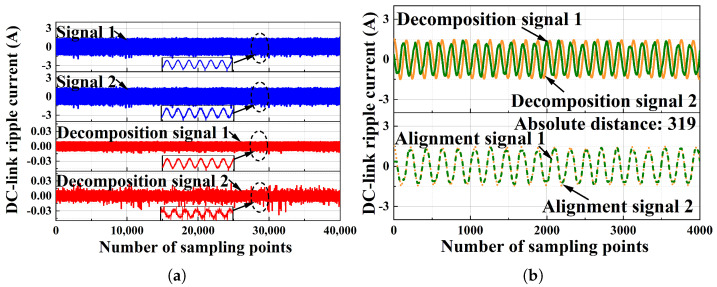
The experimental results of the signal: (**a**) the results of ICA output; (**b**) the results of DTW.

One unique advantage of our method is robustness. In order to verify this, experiments were conducted under external environmental disturbance, which would most likely lead to false alarms for arc faults. To verify whether our method can distinguish between external environmental disturbance and arc faults, normal signals were collected under an external environmental disturbance scenario and input to the method. The experimental results are shown in Figure 11, where Figure 11a shows the results of the ICA output and Figure 11b shows the results of DTW. It can be seen in Figure 11 that the absolute distance is 319, which is less than the threshold of 1000 and does not mistake external environmental disturbance signals for arc fault signals. This is because the disturbance from the external environment is Gaussian in terms of a high-frequency manner. According to the analysis in Section 4, the Gaussian signal cannot be decomposed by ICA. This showcases that our method is robust against Gaussian noise such as in terms of grid energy fluctuation. Furthermore, while dynamic load and weather changes impact the accuracy of traditional methods like CNNs, our method is unaffected by these factors due to its basis in source independence.

### 4.4. Comparative Study

In this subsection, a comparative study with some classical arc fault detection methods is conducted under different scenarios. The baseline methods include

1. Fast Fourier Transform (FFT) [6]: FFT is a feature-based detection method which extracts arc frequency domain features to detect arc faults.
2. Discrete Wavelet Transform (DWT) [7]: DWT is a feature-based detection method which extracts arc time–frequency domain features to detect arc faults.
3. Convolutional Neural Network (CNN) [17]: A CNN is a data-based detection method which uses convolution neural networks to identify arc faults.

Regarding the method (DA-DCGAN), it is highly complex and cannot be deployed on low-cost microprocessors for practical engineering applications.

The area under the curve (*AUC*) is used as a comparison metric, that is

(22) $$\begin{array}{c} AUC=\frac{\sum_{ins[s]\in pos}{\mathrm{rank}}_{ins[s]}-\frac{M\times (M+1)}{2}}{M\times N}. \end{array}$$

where ${\mathrm{rank}}_{ins[s]}$ represents the position number of the $s_{th}$ sequence sorted by probability, *M* is the number of positive samples, *N* is the number of negative samples, and ins[s]∈pos represents the ordinal sum of the positive samples.

**Figure 12:**
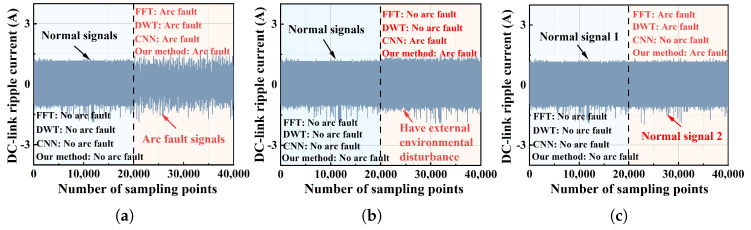
The detection results: (**a**) the detection results of each method in the case of arc faults; (**b**) the detection results of each method under external environmental disturbance; (**c**) the detection results of each method at different moments.

Comparative experiments were conducted under the three following scenarios: 1. arc faults; 2. external environmental disturbance; and 3. normal signals but at different moments. Figure 12a shows the results of arc faults; it can be seen that four methods can be detected correctly. This is because the arc faults are significant and can be easily detected. Figure 12b shows the results of external environmental disturbance. It can be seen that the FFT and DWT methods failed to detect arc faults. This is because there are high-frequency components superimposed when an external environmental disturbance is present, which may lead to a false alarm if frequency-based methods are used. Figure 12c shows the results of normal signals at different moments. It can be seen that the CNN method causes a fault alarm. This is because the accuracy of the CNN method depends on the quality of data and is limited by low interperability and low generalization.

Figure 12 demonstrates that the core idea of our method is fundamentally different from other methods. Both FFT and DWT methods detect arc faults based on local features of the arc [26]. This study uses independent component analysis (ICA) to decompose signals: DC current signals with an arc fault can be separated into two independent components, while normal signals cannot be decomposed into such independent components. Our method focuses on global features of the arc, achieving a significantly lower false alarm rate compared to FFT and DWT. As for the CNN, it requires large amounts of data to achieve high accuracy. However, arc fault data are difficult to obtain, resulting in poor interpretability of this method. In contrast, our method is based on the physical characteristics of arc faults, achieving higher accuracy than the CNN.

The Table 1 compares the performance characteristics of four arc fault detection methods. In terms of detection time, our method requires 220 μs, which is slightly slower than FFT (50 μs) but comparable to DWT (200 μs) and much faster than the CNN (5 ms). Although the detection time of our method is not the fastest, it is acceptable in practical arc fault detection tasks.

## 5. Conclusions

In this paper, a novel arc fault detection method for PV systems is proposed that relies on an arc’s global features instead of local ones. Our method is also applicable to both grid-connected and off-grid microgrid systems. This is because our method can detect DC-side arc faults within a PV inverter, and PV inverters are suitable for both grid-connected and off-grid microgrid systems. The core idea of the proposed method is that the physical mechanisms of the arc signal and the normal ones are considered to be so different that they are thought to be generated by two independent sources. Based on this insight, an ICA-DTW method is proposed for DC series arc fault detection in PV systems. The proposed method firstly employees ICA to decompose the solar inverter’s DC current signals and then uses DTW to determine the independent level of ICA’s output. Theoretical analysis and experimental results show that some traditional detection methods (e.g., FFT and DWT), which rely on the time-domain or frequency-domain features of arcs, fail to provide a comprehensive characterization of arc faults. This can lead to detection failures, especially in noisy environments. As for methods such as CNNs, they require large amounts of high-quality data for training, making high accuracy difficult to achieve. In contrast, our method is based on the global features of arc faults and does not require massive datasets, significantly enhancing the accuracy and robustness of arc fault detection. This is expected to play a substantial role in practical applications. The impact of specific parameter settings in various arc fault detection methods on the detection results will be the focus of our subsequent research.

## Figures and Tables

**Table 1:** Performance comparison of different arc fault detection methods.

Method Type	Parameter Configuration	Detection Time
FFT	4096 sampling points	50 μs
DWT	6-layer db4 decomposition	200 μs
CNN	4-layer, 20 neurons/layer	5 ms
Our method	4096 sampling points	220 μs

## Data Availability

The data that support the findings of this study are available in the figures and tables of this article.
